# A Josephson radiation comb generator

**DOI:** 10.1038/srep12260

**Published:** 2015-07-20

**Authors:** P. Solinas, S. Gasparinetti, D. Golubev, F. Giazotto

**Affiliations:** 1 SPIN-CNR, Via Dodecaneso 33, 16146 Genova, Italy; 2 Department of Physics, ETH Zürich, CH-8093 Zürich, Switzerland; 3 Low Temperature Laboratory (OVLL), Aalto University School of Science, P.O. Box 13500, 00076 Aalto, Finland; 4 Institute of Nanotechnology, Karlsruhe Institute of Technology, D-76021 Karlsruhe, Germany; 5 NEST, Instituto Nanoscienze-CNR and Scuola Normale Superiore, I-56127 Pisa, Italy

## Abstract

We propose the implementation of a Josephson Radiation Comb Generator (JRCG) based on a dc superconducting quantum interference device (SQUID) driven by an external magnetic field. When the magnetic flux crosses a diffraction node of the critical current interference pattern, the superconducting phase undergoes a jump of *π* and a voltage pulse is generated at the extremes of the SQUID. Under periodic drive this allows one to generate a sequence of sharp, evenly spaced voltage pulses. In the frequency domain, this corresponds to a comb-like structure similar to the one exploited in optics and metrology. With this device it is possible to generate up to several hundreds of harmonics of the driving frequency. For example, a chain of 50 identical high-critical-temperature SQUIDs driven at 1 GHz can deliver up to a 0.5 nW at 200 GHz. The availability of a fully solid-state radiation comb generator such as the JRCG, easily integrable on chip, may pave the way to a number of technological applications, from metrology to sub-millimeter wave generation.

Optical frequency combs have been a major research trend of the last decade^1^. The possibility to generate higher harmonics starting from a fundamental one has made it possible to extend the accuracy of the atomic clocks from the radio to the optical frequency region, leading to breakthroughs in optical metrology^2^, high precision spectroscopy^3^,^4^ and telecommunication technologies^1^,^5^. Here we show that a similar-in-spirit harmonic generator can be implemented with a dc superconducting quantum interference device (SQUID) subject to a time-dependent magnetic field. Driven by the field, the superconducting phase difference across the SQUID undergoes jumps of *π*, which are associated to a sequence of sharp voltage pulses. This pulse sequence translates into a radiation comb in frequency domain, thereby realizing a Josephson radiation comb generator. This device could have applications extending from the precision frequency measurement (as in the optical analogue) to the use as a sub-millimiter wave generation. The main advantages are the possibility to fabricate it on-chip and its integrability within the standard electrical circuits.

Our proposal for a JRCG is based on a dc SQUID (see Fig. 1a), consisting of two Josephson junctions arranged in parallel in a superconducting loop. The SQUID is biased by a constant current *I*_*B*_ and it is driven by an external, time-dependent magnetic flux Φ. Here we assume the inductance of the loop to be negligible with respect to the Josephson inductance of the junctions. Due to the first Josephson relation^6^, the current (*I*_*J*_) vs phase relation of the SQUID reads


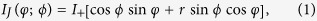


where *φ* = (*φ*_1_ + *φ*_2_)/2, *ϕ* = *π*Φ/Φ_0_ (Φ_0_ ≃ 2 × 10^−15^ Wb is the flux quantum), *I*_+_ = *I*_*c*1_ + *I*_*c*2_, *φ*_*i*_ and *I*_*ci*_ (*i* = 1, 2) are the phase across and the critical current of the *i*-th junction, respectively, and *r* = (*I*_*c*1_ − *I*_*c*2_)/(*I*_*c*1_ + *I*_*c*2_) expresses the degree of asymmetry of the interferometer. Equation (1) describes the well-known oscillations of the SQUID critical current *I*_*c*_(*ϕ*) = max_*φ*_*I*_*J*_(*φ*; *ϕ*) as a function of the magnetic flux, with minima occurring at integer multiples of Φ_0_/2 (see Fig. 1b)^6^, and it already contains the main feature of the effect we want to discuss. Let us consider the behavior of the phase *φ* as Φ crosses a critical-current minimum and take a symmetric SQUID (*r* = 0) for simplicity. If the biasing current is fixed, then we see from Eq. (1) that a change of sign in cos *ϕ* must be accompanied by a change of sign in sin *φ* in order for the current to maintain its direction. This change of sign is accomplished by a phase jump of *π*^7^,^8^,^9^, which, owing to the second Josephson relation^6^, results in a voltage pulse *V*(*t*) across the SQUID.

For a quantitative characterization of the phase jumps, we need to study the dynamics of the phase. To do so, we rely on the resistively and capacitively shunted Josephson junction (RCSJ) model^6^. We model the SQUID as a capacitor *C*, a resistor *R*, and a non-linear, flux-dependent inductor *L*_*J*_ arranged in a parallel configuration (see Fig. 1c). We consider a sinusoidally-driven magnetic flux with frequency *ν* and amplitude *ε*, centered in the first node of the interference pattern, so that Φ(*t*) = Φ_0_/2[1 − *ε*cos(2*πνt*)]. As a result, the magnetic flux crosses the nodes of the interference pattern at *t* = (2*k* + 1)/4*ν*, with *k* integer. The equation for *φ* can be written in terms of the dimensionless variable *τ* = 2*πνt* as^6^


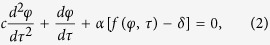


where *δ* = *I*_*B*_/*I*_+_, *c* = 2*πRCν*, *f*(*φ*,*τ*) = *I*_*J*_[*φ*; *ϕ*(*τ*)]/*I*_+_ and *α* = *I*_+_*R*/(Φ_0_*ν*).

Equation (2) is usually interpreted in terms of a fictitious phase particle moving in a tilted-washboard Josephson potential *E*_*J*_, as shown in Fig. 1d^6^. Here we restrict ourselves to small biasing current (*δ* ≪ 1), corresponding to a small tilt. Furthermore, we focus on the limits *c* ≪ 1 (overdamped regime) and |*α*| ≫ 1, as these two conditions maximize the JRCG performance (see SI).

We first consider a symmetric SQUID (*r* = 0). Then the time-dependent Josephson potential is *E*_*J*_(*t*) = ∫*I*_*tot*_*V*(*t*)*dt* = −*E*_*J*0_[*f*(*t*)cos *φ* + *δφ*] where *f*(*t*) = cos(*π*Φ/Φ_0_), 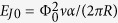, and *I*_*tot*_ = *I*_*J*_ − *I*_*B*_^6^. When *t* = 0 the potential has minima at *φ* = 2*kπ*. For *t* = 1/(4*ν*) the potential barrier vanishes and *E*_*J*_ = −*E*_*J*0_*δφ*. For *t* > 1/(4*ν*), *f*(*t*) changes sign and the potential minima occur at *φ* = (2*k* + 1)*π*. The former equilibrium points *φ* = 2*kπ* have become unstable and the system tends to move to one of the new minima, resulting in a *π*-jump in the phase. This cartoon picture helps us to pinpoint the difference between the phase jumps discussed in this work, the 2*π*-phase slips appearing in low-dimensional superconductors^10^,^11^,^12^,^13^ and the 2*π*-phase jumps used in the rapid single flux quantum (RSFQ) logic^14^,^15^. 2*π*-phase slips typically stem from thermal activation or quantum fluctuations. As for the RSFQ 2*π*-phase jumps, they are generated by a current pulse in an otherwise static potential landscape. By contrast, in the JRCG the magnitude of the jumps is *π* and the jumps have a purely energetic origin.

The numerical solution of Eq. (2) for *r* = 0 is shown in Fig. 2a. As the critical current crosses the minimum at Φ = Φ_0_/2, the phase experiences a *π*-jump and a voltage pulse is generated across the SQUID. The shape of the pulse is determined by the parameter *I*_+_*R* (see SI): the *larger I*_+_*R*, the *sharper* the voltage pulse. We notice that the presence of a finite bias current *I*_*B*_ is crucial to impose a preferred direction to the phase jumps (see also Fig. 3a). The same analysis essentially holds as well for a weakly-asymmetric SQUID (*r* ≪ 1), as long as *I*_*B*_ is strong enough to force the phase particle to roll always in the same direction. However, the junctions asymmetry brings in a key ingredient to the JRCG, which becomes apparent in the limit *I*_*B*_ → 0. Indeed, a finite asymmetry imposes an alternate pattern to the phase jumps (see Fig. 3b and SI). This realizes an ideal ac pulse source.

We can explain this behavior following the analogy with the phase particle in a time-dependent potential. For *r* ≠ 0, the position of the minima changes in time (see Fig. 3 and SI). This means that if the system starts in a minimum at *t* = 0, it is close but not in a maximum when the time-dependent potential changes sign. This small deviation from the maximum point induces the phase particle rolling and the corresponding phase jump even in absence of current bias. For a periodic drive, the particle is found alternatively on the left and on the right of the maximum; as a result, it rolls in alternate directions producing the alternate pattern of the voltage pulses. In Fig. 3b, we show the potential *E*_*J*_ vs *φ* at different time. The blue thick curve represents the position of the minima of the potential. In the absence of a current bias, the phase undergoes a sequence of positive and negative jumps resulting in the alternate voltage pulses. This device configuration realizes an ideal ac pulse source. Furthermore, the limit *I*_*B*_ → 0 corresponds to a floating device. This facilitates the integration of the JRCG in microwave-based architectures such as circuit-QED^16^,^17^,^18^.

The voltage pulses shown in Fig. 2 suggest an application similar to the frequency combs used in optics^1^. In this context, the most relevant feature becomes the sharpness of the voltage pulse, which is related to the number of harmonics generated. The sharpness is essentially determined by *I*_+_*R*, which, in turn, depends on the material properties of the Josephson junctions. While the total output power provided by a single JRCG is fairly small, it can be boosted by using an array of nominally-identical SQUIDs. A similar approach is used for the realization of the metrological standard for voltage based on the Josephson effect^19^,^20^,^21^,^22^,^23^,^24^. Before presenting our results for an array of SQUIDs, we discuss the approximations we have used in our analysis.

First of all, we have neglected the coupling between the SQUIDs via mutual inductance and/or cross capacitance and inductance of the superconducting wire. This condition, which can be realized in practice by a suitable design choice, implies that the dynamics of each SQUID is independent from the rest of the chain. As a matter of fact, due to current conservation, the current *I*_*i*_ through the *i*-th SQUID is the same and equal to *I*_*B*_: *I*_*i*_ = *I*_*B*_. For every SQUID we can write the total current as *I*_*i*_ = *I*_*i*,*S*_ + *I*_*i*,*N*_ + *I*_*i*,*C*_ = *I*_*B*_ where *I*_*i*,*S*_ = *I*_*i*,*C*_ cos(*π*Φ/Φ_0_)sin *φ*_*i*_ is the superconducting current (for *r* = 0), *I*_*i*,*N*_ = *V*_*i*_/*R*_*i*_ is the normal current and *I*_*i*,*D*_ = *C*_*i*_*dV*_*i*_/*dt* is the displacement current. In the above expressions, *V*_*i*_ and *φ*_*i*_ are the voltage and the phase across the *i*-th SQUID, *C*_*i*_ and *R*_*i*_ are the capacitance and the resistance of the SQUID, respectively. By using the Josephson voltage-phase relation we then obtain the RCSJ Eq. (2) for the *i*-th SQUID, which is independent from the other SQUIDs in the array. Given this result, it is possible to obtain the voltage at the extremes of the array by summing up the voltage of the single SQUIDs: *V*(*t*) = ∑_*i*_*V*_*i*_(*t*).

So far we have discussed the voltage produced by the JRCG in the absence of any external load. From an experimental point of view, a quantity of greater interest is the power that can be transferred to a given load *R*_*L*_. The effect of a finite load on the chain can be understood in terms of an additional current flowing through the chain. Under the assumption that all the SQUIDs are identical, the problem can still be treated exactly. It turns out (see SI) that the voltage across the load is still given by ∑_*i*_*V*_*i*_(*t*), provided an additional shunt resistor *R*_*L*_/*N* is added in parallel to each SQUID. The shunt resistance *R* in the RCSJ equation (2) must then be replaced by an effective resistance *R*_eff_ = *RR*_*L*_/(*R*_*L*_ + *NR*). We find that the delivered power *P* = *V*(*t*)^2^/*R*_*L*_ scales as *N*^2^ as long as *N* ≪ *R*_*L*_/*R*, while in the opposite limit *N* ≫ *R*_*L*_/*R* it scales as *N*.

A further assumption we have made is that the emitted radiation propagates instantly across the device. Such lumped-element model is certainly appropriate for a short SQUID chain but will eventually break down as the total length of the chain approaches the wavelength of the emitted radiation. In that limit, the chain must be regarded as a distributed element and we generally expect the frequency comb to be distorted by wave-interference effects. The relation between the minimum wavelength *λ*_min_ and the chain length *L* relation for the validity of the lumped-element model is (see SI): *λ*_min_ ≥ *L*/2. However, even when this condition is no longer satisfied, a suitable choice of the effective distance between the SQUIDs can ensure constructive interference at a specific frequency. This feature can be exploited to operate the device at higher frequencies and/or with higher output.

Now, we are in position to present the predicted performance of the device. In Fig. 4 we show the calculated JRCG power spectrum *P* vs frequency Ω (see SI) for two driving frequencies and for different junctions and symmetry parameters. Figure 4a displays the behavior of a chain of *N* = 50 symmetric Nb/AlOx/Nb SQUIDs^25^ with a 100 MHz drive. The parameters are the same as those in Fig. 2a. The sharp pulses determine the broad range of the emitted radiation, up to several hundreds of harmonics. At 20 GHz (see the inset of Fig. 4a) the JRCG provides an output power of ~1 pW. This power level can be detected, for instance, by coupling the device to a transmission line and feeding the signal to a commercial spectrum analyzer.

In order to achieve sharp pulses at higher frequencies, one needs to use a superconductor with a larger characteristic voltage (*I*_+_*R*). In such a way, one can drive the SQUID at higher frequencies. In Fig. 4b we show the results expected for a symmetric YBCO SQUID series^24^,^26^,^27^ at 1 GHz drive. YBCO Josephson junctions provide a large superconducting gap with a characteristic voltage *I*_+_*R* ≈ 10 mV and possess a negligible intrinsic capacitance^27^. Due to the larger driving frequency, the emitted signal at 200 GHz is still sizable, reaching an output power of a fraction of nW (Fig. 4b, inset). Such a signal is already in the far infrared range, which has seen a substantial research development in the last two decades due to countless technological applications. In this frequency range, the radiated signal can be coupled to free space through, for instance, an antenna coupled to the SQUID electrodes^28^,^29^. The power spectrum is similar for an asymmetric YBCO SQUID chain (Fig. 4c). The main differences lie in the presence of odd harmonics only and in a smaller output power at high frequency (around 0.2 nW at 200 GHz).

The device has room for optimization. The delivered power at high frequency can be increased by suitable array design, i.e., either by using different materials or optimizing the inter-SQUID distance, or by using parallel configuration of JRCGs (see SI).

We have analyzed the effect of thermal noise at 4.2 K on the device performance (see SI). Noise is expected to be the most harmful in the vicinity of the phase jumps, as the potential barrier is the most shallow there (see Fig. 1d). However, our numerical calculations show that its effect is negligible for the parameters used in Fig. 4. Thermal noise could play a role at slow driving frequencies because it is easier to induce undesired transitions when the the potential barrier is shallow. However, this effect can be counteracted by increasing the current *I*_*B*_ to impose a privileged direction to the dynamics (see SI). In the array configuration, particular care must also be taken to avoiding random flux offsets, due to, for instance, trapped vortices. Such offsets would cause the SQUIDs to switch at different times and thereby contribute to the smearing of the voltage comb features. Similar detrimental effects can be produced by imprecision in the SQUID fabrication, i.e., in the asymmetry parameter and SQUID area.

The proposed JRCG is within the reach of state-of-the-art nanofabrication technology. SQUID arrays with an asymmetry dispersion of the order of ~0.05–1% can be fabricated with standard lithographic techniques. Furthermore, a single on-chip superconducting line can be used to drive the magnetic fluxes of a SQUID array in a synchronized manner and with ns time resolution. The delivered power at high frequency may be increased beyond our estimates by fabrication and/or design, for instance, by using different materials or by operating more JRCGs in a parallel configuration (see SI).

In summary, we have proposed a Josephson radiation comb generator based on a flux-driven SQUID array. Based on our preliminary analysis, its implementation seems realistic and may pave the way to a number of applications, from low-temperature microwave electronics to on-chip sub-millimiter wave generation.

## Methods

To obtain the voltage power spectrum we first calculate the Fourier transform of the voltage *V*(*t*)


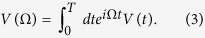


The power spectral density (PSD) is then


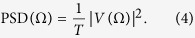


The power *P* discussed in the main text is calculated by integrating the PSD around the resonances *kν* (where *ν* is the monochromatic drive frequency) and dividing for a standard load resistance of 50 Ohm. This is the power we would measure *at a given resonance frequency* with a bandwidth exceeding the linewidth of the resonance.

## Additional Information

**How to cite this article**: Solinas, P. *et al*. A Josephson radiation comb generator. *Sci. Rep*. **5**, 12260; doi: 10.1038/srep12260 (2015).

## Supplementary Material

Supplementary Information

## Figures and Tables

**Figure 1:**
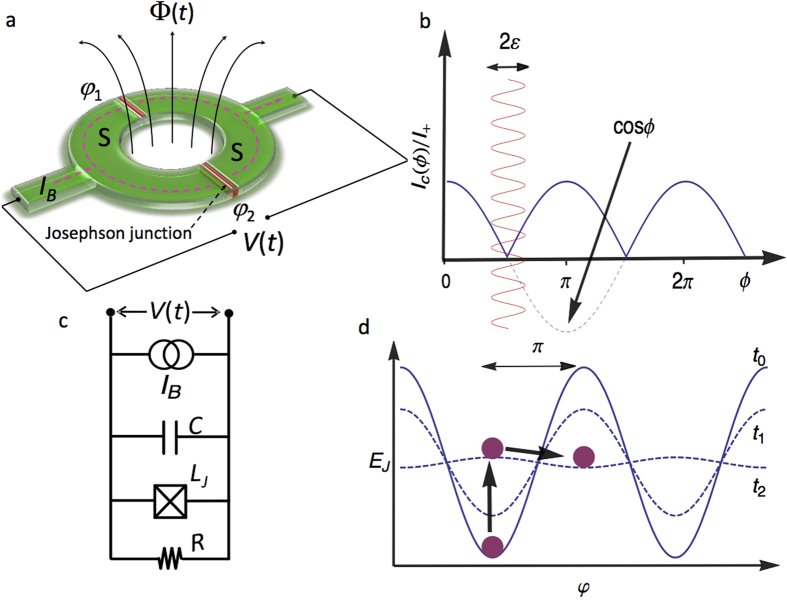
The Josephson radiation comb generator. (**a**) A current-biased, flux driven SQUID generates a time-dependent voltage *V*(*t*). The SQUID consists of two superconducting electrodes *S* (green) connected by two Josephson junctions (red). *φ*_*i*_ is the phase across the *i*-th junction, *I*_*B*_ is the constant current bias and Φ(*t*) is the time-dependent magnetic flux. (**b**) Normalized critical current *I*_*c*_(*ϕ*)/*I*_+_ versus normalized magnetic flux *ϕ* = *π*Φ/Φ_0_ for a symmetric SQUID (solid line). The *ϕ*-dependent term cos *ϕ* is also plotted as a dashed line. The phase *φ* across the SQUID undergoes a *π* jump whenever *ϕ* crosses an interference node, due to the change in sign of cos *ϕ*. Phase jumps can be induced by modulating the flux in time around an interference node with a small amplitude *ε* (red line). (**c**) RCSJ model circuit for the SQUID, with resistance *R*, Josephson inductance *L*_*J*_, and capacitance *C*. (**d**) Time-dependent tilted-washboard potential for the RCSJ model. The potential is plotted at the initial time (*t*_0_ = 0), at an intermediate time (*t*_1_ = 0.17/*ν*, where *ν* is the frequency of the modulation), and just after the vanishing of the potential barrier (*t*_2_ = 0.26/*ν*). The phase particle (purple ball) starts in an energetic minimum at *φ* = 2*kπ*. At times later than 1/(4*ν*), the position of the particle becomes unstable, leading to a phase jump to the nearest minimum at *φ* = (2*k* + 1)*π*. The direction of the jump is determined by the washboard tilt *δ* = *I*_*B*_/*I*_+_, with *δ* ≪ 1.

**Figure 2:**
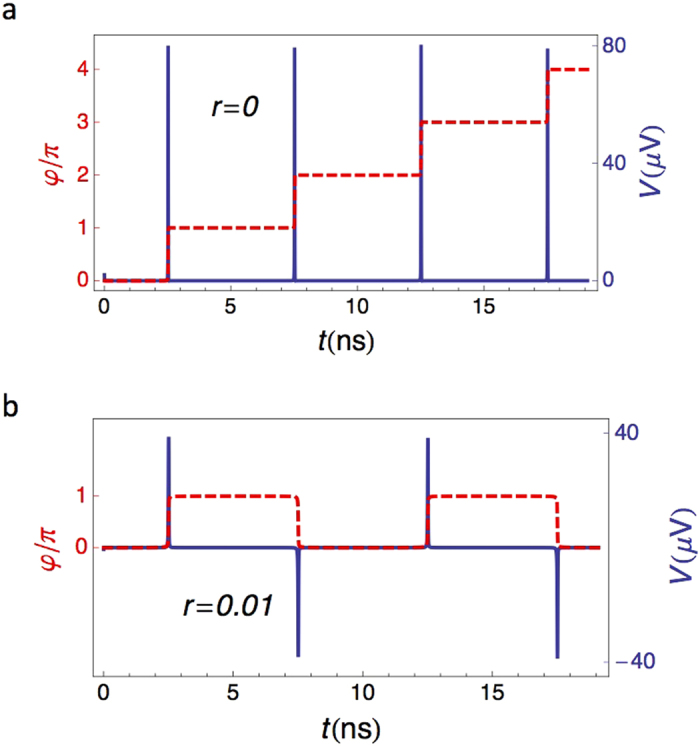
Phase jumps and voltage comb. Time evolution of the phase *φ* (dashed line, left axis) and corresponding voltage *V* developed across the SQUID (solid line, right axis). Voltage pulses are generated at times (2*k* + 1)/4*ν* (with *k* integer), when an interference node is crossed. The evolution is shown for a symmetric SQUID (**a**) with *r* = 0 and *I*_*B*_ = 10^−3^*I*_+_, and for an asymmetric SQUID (**b**) with *r* = 0.01 and *I*_*B*_ = 0. In (**a**) the current bias determines the direction of the phase jumps. In (**b**) the SQUID asymmetry induces alternate phase jumps even in the absence of an external current bias. The driving frequency is *ν* = 100 MHz and the amplitude is *ε* = 0.9. The SQUID parameters are typical for a Nb/AlOx/Nb junction^25^, with *R* = 10 Ohm, *I*_+_ = 0.2 mA and *I*_+_*R* = 0.2 mV. The corresponding capacitance would be of the order of =10 fF and has been neglected.

**Figure 3:**
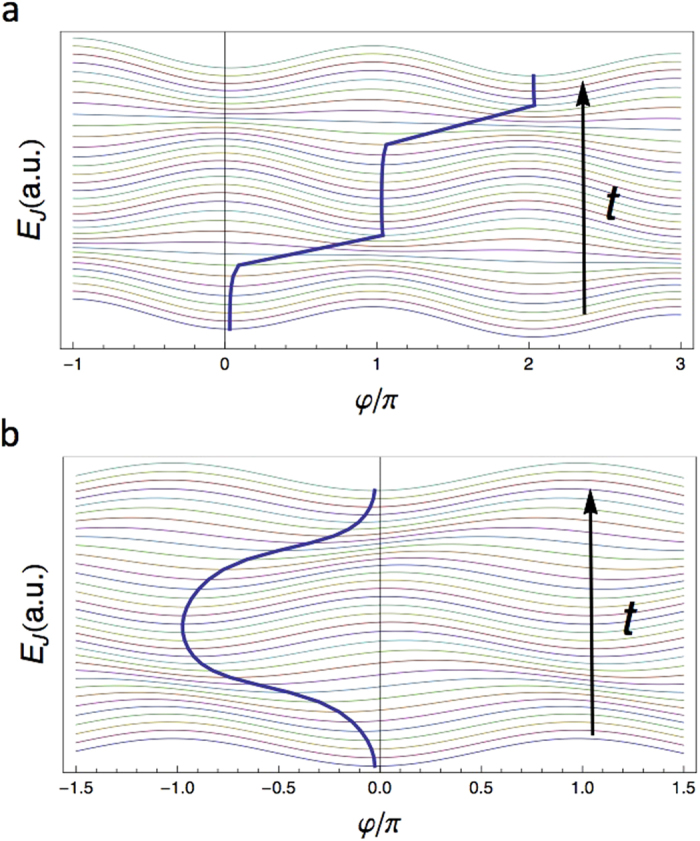
Tilted-washboard potential for symmetric and asymmetric SQUID. The potential landscape *E*_*J*_(*φ*) is plotted at increasing times *t*. The traces are stacked by a constant offset on the vertical axes. A blue, thick curve tracks the position of a local minimum of the potential at different times. (**a**) Symmetric SQUID, i.e., *r* = 0, with tilt *δ* = 0.3. (**b**) Asymmetric SQUID, with *r* = 0.5 but *δ* = 0. For presentation purposes, we have chosen larger values of *r* and *δ* than those giving the best performance for the device (see Fig. 4).

**Figure 4:**
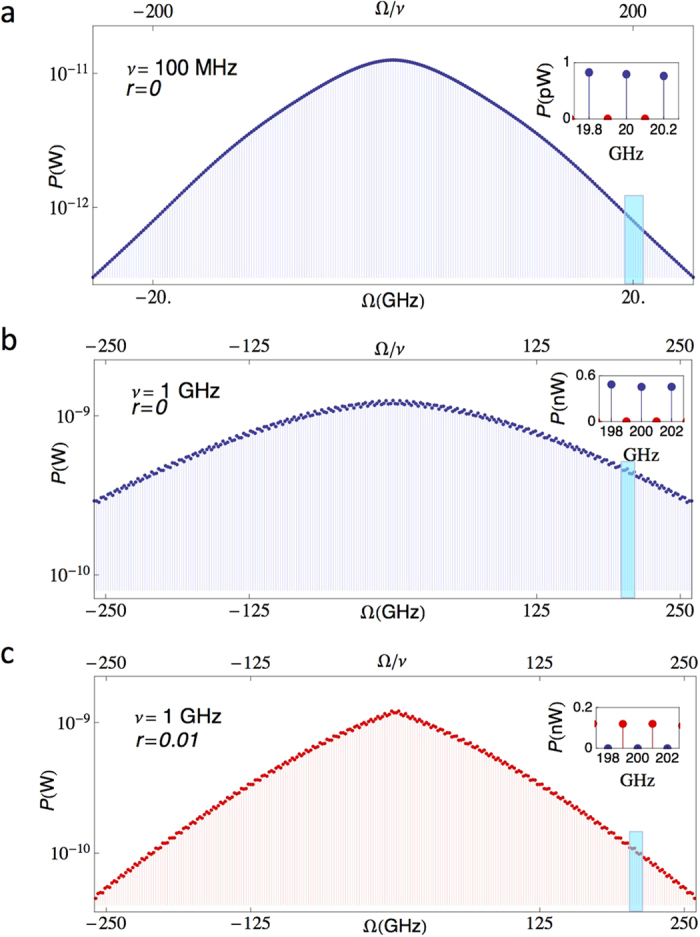
Power spectrum of the Josephson radiation comb generator. We consider a chain of *N* = 50 SQUIDs coupled to a 50Ω load. To emphasize the behavior at high frequency we use a logarithmic scale in the main panels but keep the linear scale in the insets. The cyan regions correspond to the insets. (**a**) Power spectrum *P* vs frequency Ω for a symmetric (*r* = 0) Nb/AlOx/Nb SQUID chain, driven at *ν* = 100 MHz. The SQUID parameters are the same as in Fig. 2. Due to the presence of the load, each SQUID sees an effective resistance *R*_eff_ ≈ 1 Ohm. The output power at 20 GHz (200-th harmonic) is about 1 pW (see Inset). (**b**) *P* vs Ω for a symmetric (*r* = 0), high-critical temperature YBCO SQUID chain, with *ν* = 1 GHz, *I*_+_*R* = 10 mV, *R*_eff_ = 1 Ohm and *I*_*B*_ = 10^−3^*I*_+_^26^,^27^. At 200 GHz (200-th harmonic) *P* is about 0.5 nW (see Inset). (**c**) *P* vs Ω for an asymmetric (*r* = 0.01) YBCO SQUID chain, with *ν* = 1 GHz, *I*_+_*R* = 10 mV and *I*_*B*_ = 0. In all panels, the drive is such that Φ(*t*) oscillates around the first interference node (Φ_0_/2) with elongation *ε* = 0.9. Blue and red dots are used to plot the even and the odd harmonics, respectively. In (**a,b**) only the even harmonics are present due to the comb-like shape of the voltage pulses. In (**c**) due to the alternating direction of the voltage pulses, only the odd harmonics are present. The junction capacitance has been neglected in all calculations as it does not affect the dynamics.
