# Supplementary material for: A Josephson radiation comb generator
Source: Sci Rep. 2015 Jul 20;5:12260. doi: 10.1038/srep12260 (PMC4507479; doi:10.1038/srep12260)
Supplement: Supplementary Information [file srep12260-s1.pdf]

Supplementary Information
A Josephson radiation comb generator

[1] P. Solinas, [2,3] S. Gasparinetti, [3,4] D. Golubev and [5,⋆] F. Giazotto

[1] *SPIN-CNR, Via Dodecaneso 33, 16146 Genova, Italy*

[2] *Department of Physics, ETH Zürich, CH-8093 Zürich, Switzerland*

[3] *Low Temperature Laboratory (OVLL), Aalto University School of Science, P.O. Box 13500, 00076 Aalto, Finland*

[4] *Institute of Nanotechnology, Karlsruhe Institute of Technology, D-76021 Karlsruhe, Germany*

[5] *NEST, Instituto Nanoscienze-CNR and Scuola Normale Superiore, I-56127 Pisa, Italy*

Correspondence should be addressed to - FG.

⋆ e-mail: giazotto@sns.it

I. SOLUTION OF THE RCSJ EQUATION IN A DC SQUID

We consider a dc SQUID composed by two Josephson junctions and subject to a magnetic flux Φ. The total current though the SQUID is $I_J = I_{c1}\sin\varphi_1 + I_{c2}\sin\varphi_2$, where I_{ci} and φ_i are the critical current and the phase across the i-th junction, respectively. Because of the flux quantization constraint, it follows that $(\varphi_1 - \varphi_2)/2 = \pi\Phi/\Phi_0$. Introducing the phase across the SQUID $\varphi = (\varphi_1 + \varphi_2)/2$, we get

$$I_J[\varphi; \phi(\tau)] = I_+[\cos\phi \sin\varphi + r\ \sin\phi\cos\varphi]\,, \tag{1}$$

where $\phi = \pi\Phi/\Phi_0$, $I_+ = I_{c1} + I_{c2}$, $r = (I_{c1} - I_{c2})/(I_{c1} + I_{c2})$ and $\Phi_0 \simeq 2 \times 10^{-15}$ Wb is the flux quantum.

Starting from the RCSJ model [1], we can write an equation of motion for the phase φ as

$$\frac{\hbar C}{2e}\ddot{\varphi} + \frac{\hbar}{2eR}\dot{\varphi} + I_+ f(\varphi, t) = I_B \tag{2}$$

where C is the capacitance, R is the total shunting resistance of the SQUID, I_B is the external biasing current and $f(\varphi, t) = I_J[\varphi; \phi(t)]/I_+$. We rescale the above equation in terms of the driving frequency ν: $\tau = 2\pi\nu t$. Using $\hbar/(2e) = \Phi_0/(2\pi)$, we obtain

$$c\frac{d^2\varphi}{d\tau^2} + \frac{d\varphi}{d\tau} + \alpha[f(\varphi, \tau) - \delta] = 0, \tag{3}$$

where $\delta = I_B/I_+$, $c = 2\pi RC\nu$ and

$$\alpha = \frac{I_+ R}{\Phi_0 \nu}. \tag{4}$$

A. Analytical solution for $I_B = 0$

Let us consider the case of a symmetric dc SQUID ($r = 0$), overdamped junctions ($c \approx 0$) and zero current bias ($\delta = 0$). Then (3) reduces to

$$\frac{d\varphi}{d\tau} + \alpha F(\tau)\sin\varphi = 0, \tag{5}$$

where $F(\tau) = \cos[\phi(\tau)]$. We notice that if the initial condition is $\varphi(0) = \varphi_0 = k\pi$, the above equation has trivial dynamics $\varphi(t) = 0$. This means that, even if small, we cannot neglect the influence of the $\ddot{\varphi}$ term. However, if $\varphi \neq k\pi$, we can effectively neglect the capacitive contribution and solve analytically the differential equation (3) to obtain

$$\varphi(t) = 2\arctan\Big[\exp\Big(-\alpha\int_0^t d\tau F(\tau)\Big)\tan\frac{\varphi_0}{2}\Big]. \tag{6}$$

We suppose that $|\alpha| \gg 1$ (and $\alpha > 0$). If $\int_0^t d\tau F(\tau)$ assumes positive and negative values, the argument of the arctangent increases or decreases exponentially depending on its sign. For simplicity, we consider $\int_0^t d\tau F(\tau) < 0$ and the case of small φ_0. For $\varphi_0 > 0$, $\varphi(t)$ exponentially reaches π, and for $\varphi_0 < 0$, the evolution is similar but $\varphi(t)$ varies between φ_0 and $-\pi$. Therefore, in both cases we have an exponential π jump of the phase but its direction is determined by the initial phase φ_0. The rate of the exponential jump is determined by I_+R: the larger I_+R, the sharper the voltage pulse.

Recalling the phase particle analogy discussed in the main text, with $c = 0$ if the phase is initially in the minimum $\varphi_0 = 2k\pi$ it will remains in the same point even when it becomes an unstable maximum. A small shift of the initial condition induces an exponential dynamics since at $t = 1/(4\nu)$ the phase is close to (but not on) a potential maximum. The direction of this shift (and, in the case discussed, the sign of φ_0) determines the direction of the fall and the jump of the phase.

The change in time of φ in Eq. (6) is associated to a voltage

$$\frac{2e}{\hbar}V(t) = \frac{-2\pi\nu\alpha\ F(t)\sin\varphi_0}{\sin^2\left(\frac{\varphi_0}{2}\right)e^{-\alpha\int_0^t f(\tau)\,d\tau} + \cos^2\left(\frac{\varphi_0}{2}\right)e^{\alpha\int_0^t f(\tau)\,d\tau}} \tag{7}$$

(Notice that we have gone back the real time $t = \tau/(2\pi\nu)$ unit.)

For a small oscillation around the first diffraction node it is possible to have an analytical expression for $V(t)$. In this case, $\cos(\pi\Phi/\Phi_0) \approx (\pi/2)\epsilon\cos(2\pi\nu t)$. After a straightforward calculation, we approximate the function for small times, i.e., $t \ll 1/\nu$ to obtain

$$V(t) = -\frac{\pi}{2}\alpha\nu\Phi_0\epsilon\cosh^{-1}\left(\frac{\pi\alpha\epsilon t\nu}{2} + \rho_0\right) \tag{8}$$

where $\rho_0 = \log\left[\tan(\varphi_0/2)\right]$. The above expression has an exponential behavior (increasing and decreasing) when the system crosses a diffraction node.

The mean value of the voltage square, which is related to the delivered power, is given by $\langle V^2(T)\rangle = (1/T)\int_0^T dt V^2(t)$, where T is the averaging time. Taking into account Eq. (4) and for long average time $T \gg 2/(\pi\alpha\epsilon\nu)$, we obtain

$$\langle V^2(T)\rangle = \frac{\Phi_0\epsilon I_+R}{4T}\tanh\left(\frac{\pi\alpha\epsilon T\nu}{2} + \rho_0\right) \approx \frac{\Phi_0\epsilon I_+R}{4T}. \tag{9}$$

B. Solution in presence of a current bias

We consider now the case of a small biasing current I_B in a symmetric SQUID ($r = 0$). By "small biasing current", we mean that its effect must be negligible with respect to the

driven dynamics, i.e., $\delta \ll \alpha$, but must dominate the capacitor dynamics, i.e., $\delta \gg c$.

When $\varphi \approx k\pi$, the Josephson current contribution $\alpha F(\tau)\sin\varphi$ is small and the dynamics is determined only by I_B. Equation (3) reduces to

$$\frac{d\varphi}{d\tau} - \alpha\delta = 0. \tag{10}$$

Away from $\varphi \approx k\pi$, the Josephson current contribution dominates and, therefore, the equation for motion approximatively reads

$$\frac{d\varphi}{d\tau} + \alpha F(\tau)\sin\varphi(\tau) = 0. \tag{11}$$

Since the behavior of $\varphi(t)$ under these two different dynamics is drastically different (linear versus exponential change), we can tune the system parameters in order to effectively separate the two regimes governed by Eqs. (10) and (11) and generate the sequence of exponential phase jumps.

In other terms, the presence of a current bias has two effects. The first is to transport the system away from the region $\varphi \approx k\pi$ in which the drive contribution is small and the dynamics is dominated by the capacitive term. The second is to breaks the symmetry of the system inducing the jumps always in the same direction.

The effect of a current bias can be interpreted in terms of the tilted-washboard potential discussed in the main text.

C. Asymmetric SQUID case

From Eq. (1), the energy potential reads (we neglect the current bias and set $\delta = 0$) [1]

$$E_J(t) = -\cos\phi\cos\varphi + r\sin\phi\sin\varphi. \tag{12}$$

To find the position of the maxima and the minima we derive $E_J(t)$ with respect to φ and equal it to zero. The corresponding equation reads $\tan\varphi = r\tan\phi$. For $r = 0$, we see that the values of φ satisfying the above equation do not depend on time. On the contrary, since $\phi = \pi\Phi/\Phi_0$, they depend on time through Φ for any $r \neq 0$ as discussed in the main text. For a periodic drive, the particle is found alternatively on the left and on the right of the maximum and it rolls in alternate directions producing the alternate pattern of the voltage pulses.

II. FINITE-SIZE EFFECTS

We consider a long chain of SQUIDs located at $x = x_k$, $(k = 1, 2, ..., N)$ and coupled to a transmission line of length L. We assume that the first SQUID is at point $x_0 = 0$ and that $x_k = ka$, where a is the distance between the SQUIDs. Every SQUID emits radiation with the spectrum $v(\omega)$. The time dependent voltage drop across it is $V(t) = \int d\omega/(2\pi)\exp(-i\omega t)v(\omega)$. In case of periodic drive one should have $v(\omega) = \sum_n V_n\delta(\omega - n\omega_0)$, where ω_0 is the driving frequency. Then the voltage at the end of the transmission line, i.e., at $x = L$, can be estimated as a sum of the signals coming from each SQUID:

$$V(t, L) = \sum_{k=0}^{N} \int \frac{d\omega}{2\pi} v(\omega) e^{-i\omega[t-(L-x_k)/c]} \tag{13}$$

where c is the speed of electromagnetic waves propagating along the transmission line. For simplicity, we assumed that the speed is the same both in the transmission line and in the chain of SQUIDs. Performing summation over k in Eq. (13) and considering that $N \gg 1$, one finds

$$\begin{aligned} V(t, L) &= \int \frac{d\omega}{2\pi} v(\omega) \frac{1 - e^{-iN\omega a/c}}{1 - e^{-i\omega a/c}} e^{-i\omega(t-L/c)} \\ &= \sum_n \frac{V_n}{2\pi} \frac{1 - e^{-iN\omega a/c}}{1 - e^{-i\omega a/c}} e^{-in\omega_0(t-L/c)}. \end{aligned} \tag{14}$$

Thus, the intensity of n-th harmonics at $x = L$

$$P_n(L) = |V_n(L)|^2 = |V_n|^2 \left| \frac{1 - e^{-iN\omega a/c}}{1 - e^{-i\omega a/c}} \right|^2 = P_n(0) \frac{\sin^2\left(Nn\frac{\omega_0 a}{2c}\right)}{\sin^2\left(n\frac{\omega_0 a}{2c}\right)}. \tag{15}$$

The power of the n-th harmonics scales with the number of junction as N^2 if $Nan\omega/(2c) \leq 1$. This limit can be rewritten as $L/(2\lambda) \leq 1$ with $Na = L$ and $c/(n\omega_0) = \lambda_0/n = \lambda$ is the wavelength emitted.

For the parameters used in the simulation, considering a wavelength of $\lambda = 6 \times 10^{-4}$ m corresponding to 200 GHz in Silicon, and a SQUID distance of $a = 1$ μm, we have that for $N = 50$ the array can be considered as a lumped element.

III. POWER DELIVERED TO THE LOAD RESISTOR

As discussed in the main text, current conservation through the SQUID implies that dynamics of each SQUID is independent from the rest of the chain. As a consequence, the

voltage at the extremes of the chain scales as the number N of SQUIDs. Accordingly, the intrinsic power generated by the device (that is, the power delivered to an infinite load) scales as N^2. In this section we discuss the effect of a finite load on the JRCG. Let the SQUIDs be identical and $V(t)$ be the voltage drop across each SQUID. Then the voltage at the load is $V_{\rm tot}(t) = NV(t)$ and the current flowing through it is $I_L = NV(t)/R_L$, where R_L is the (real) impedance of the load. This current flows back to the SQUID array and adds up, with opposite sign, to the bias current I_B. As a result, (2) must be rewritten as

$$\frac{\hbar C}{2e}\ddot{\varphi} + \frac{\hbar}{2eR}\dot{\varphi} + I_+ f(\varphi, t) = I_B - \frac{NV(t)}{R_L} \ . \tag{16}$$

Recalling that $V_i(t) = (\hbar/2e)\dot{\varphi}$, we obtain

$$\frac{\hbar C}{2e}\ddot{\varphi} + \frac{\hbar}{2eR_{\rm eff}}\dot{\varphi} + I_+ f(\varphi, t) = I_B \ , \tag{17}$$

with

$$R_{\rm eff} = \left(\frac{1}{R} + \frac{N}{R_L}\right)^{-1} = \frac{RR_L}{R_L + NR} \ . \tag{18}$$

The effective change in the shunt resistance modifies the SQUID dynamics. In particular, it also reduces the power $P = N^2V^2/R_L$ that can be delivered to the load. From Eq. (9), we see that for a single SQUID $\langle V\rangle^2 \propto R_{\rm eff}$. Using (18), we find that P scales as N^2 for $N \ll R_L/R$ and as N for $N \gg R_L/R$.

This analysis suggests that an increase in the output power could be gained by operating more JRCGs in a parallel configuration. If M such devices are operated in parallel and are synchronized, the backflow current is reduced by a factor $1/M$ and the effective resistance reads $R_{\rm eff} = RR_L/\left[R_L + (N/M)R\right]$.

IV. EFFECT OF THE THERMAL NOISE

To estimate the effect of the thermal noise we use the Langevin equation

$$\frac{\hbar C}{2e}\ddot{\varphi} + \frac{\hbar}{2eR}\dot{\varphi} - I_+ f(t)\sin\varphi = I_B + \xi(t), \tag{19}$$

where $\xi(t)$ is the white noise with correlation function

$$\langle \xi(t)\xi(t')\rangle = \frac{2k_BT}{R}\delta(t-t'). \tag{20}$$

We have numerically solved the associated stochastic differential equation in case of a YBCO SQUID with 1 GHz drive. We have considered a symmetric YBCO SQUID with

negligible capacitance. The noise source has been taken at temperature of 4.2 K. With these parameters, the dynamics of the SQUID shown in Fig. 1 is essentially identical to that without noise source.

The thermal noise results in a small broadening of the resonances at high frequency (see Fig. 2). The signal to noise ratio is still about 10^3 and it can be further increased by decreasing the working temperature of the device.

The effect of noise is maximum when the energy barrier is shallow and undesired transitions are most likely to occur. Therefore, we expect an increased noise influence for slow frequency drive since the system remains in a shallow barrier potential for a longer time. However, even in this situation these noise effects can be reduced by increasing I_B in order to restore the privileged direction of the dynamics.

References

[1] Tinkham, M. *Introduction to superconductivity* (Courier Dover Publications, 2012).

[2] Malnou, M. *et al.* High-Tc superconducting Josephson mixers for terahertz heterodyne detection. *J. Appl. Phys.* **116**, 074505 (2014).

[3] Rosenthal, P. & Grossman, E. N. Terahertz Shapiro steps in high temperature SNS Josephson junctions. *IEEE Trans. Microw. Theory Tech.* **42**, 707–714 (1994).

Figures (Supplementary Information)

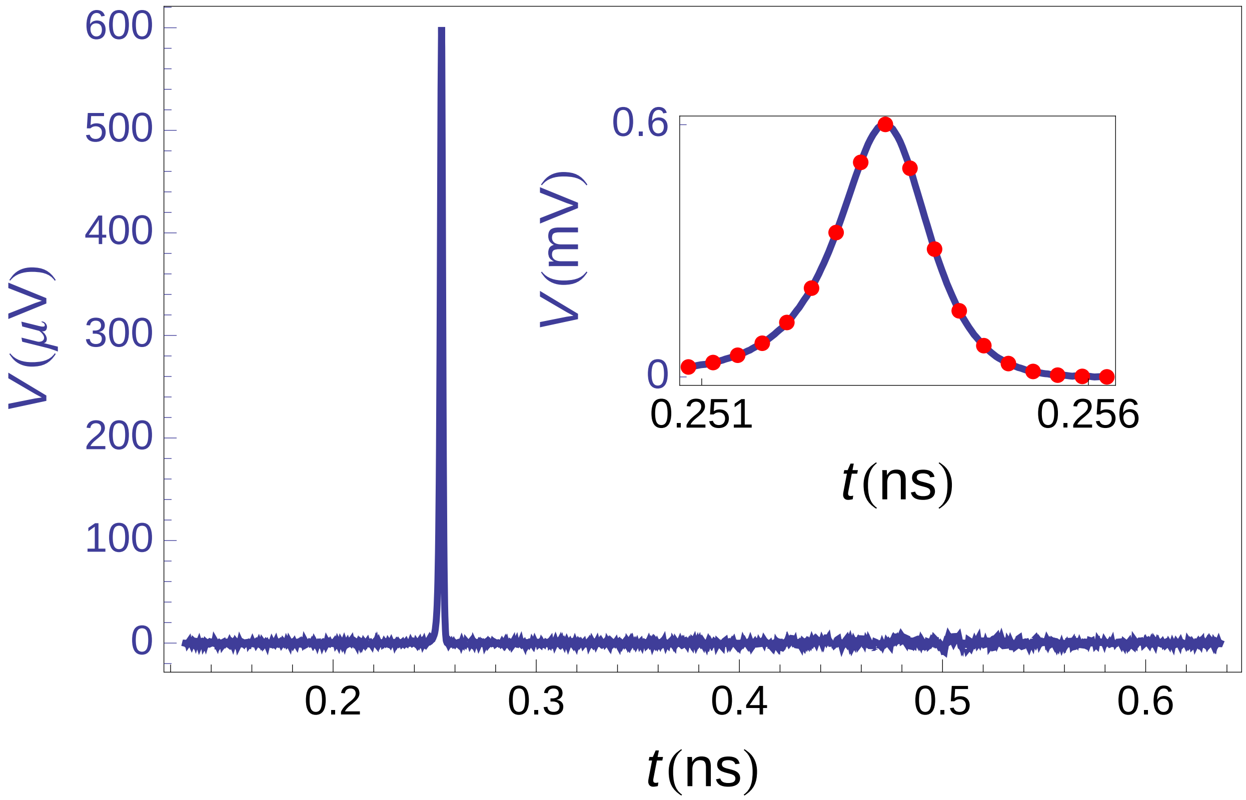

FIG. 1: **Effect of the thermal noise on the dynamics.** Voltage V developed across the SQUID (blue solid line) as a function of time t in presence of thermal noise. Here we set the driving frequency to 1 GHz. (Inset) Comparison between noisy (blue solid curve) and noise-free dynamics (red dots). The parameters are those typical of a YBCO junction, as reported in Refs. [2, 3]: $I_+R = 10$ mV and $I_B = 10^{-3}I_+$ and $\epsilon = 0.9$. The capacity is negligible and set to zero. The temperature of the thermal bath is 4.2 K.

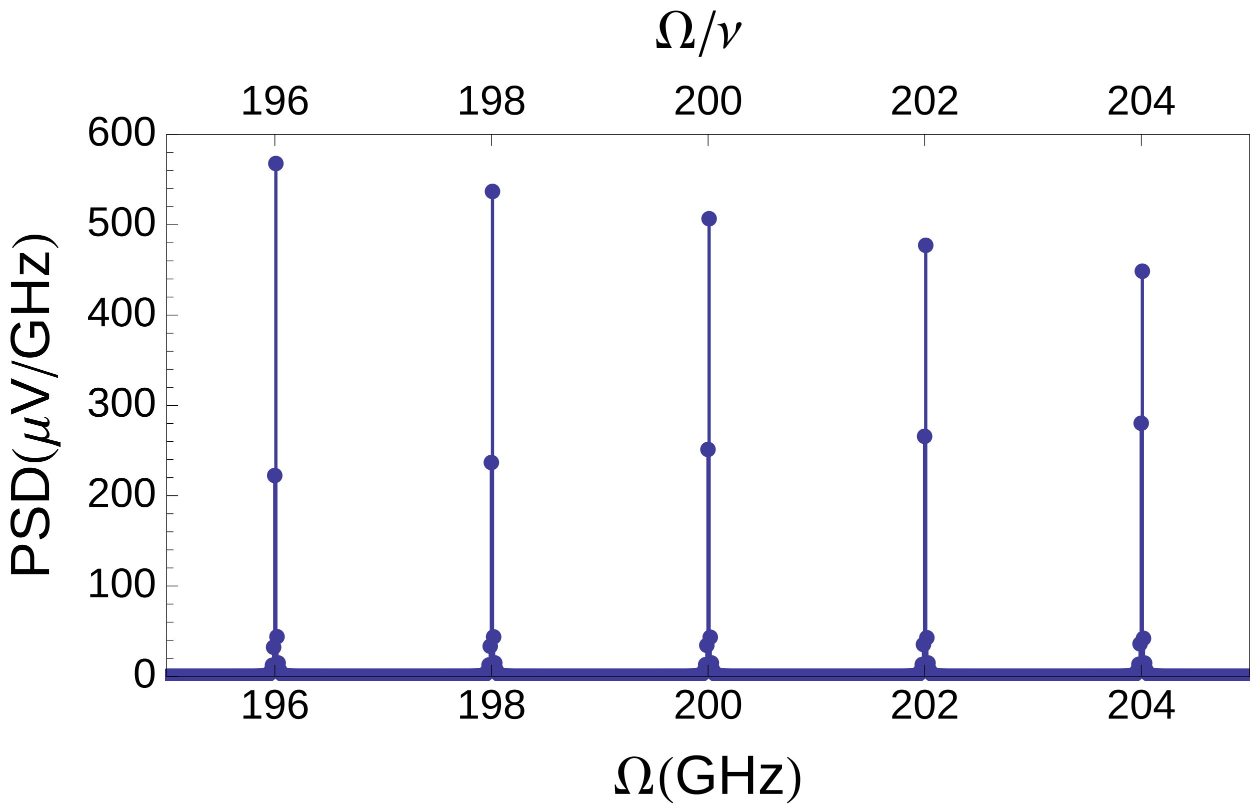

FIG. 2: **Effect of the thermal noise on power spectral density.** Power Spectral Density (PSD) for a YBCO SQUID with 1 GHz drive at high frequency. The signal to noise ratio is about 10^3. Notice that here the PSD is calculated for a single SQUID. For the calculations we set the following parameters [2, 3]: $I_+ R = 10$ mV and $I_B = 10^{-3} I_+$. The junction capacitance is typically very small, $C \sim$ fF, and has been neglected in the numerics.
